# NOTCH1 Signaling Promotes Human T-Cell Acute Lymphoblastic Leukemia Initiating Cell Regeneration in Supportive Niches

**DOI:** 10.1371/journal.pone.0039725

**Published:** 2012-06-29

**Authors:** Wenxue Ma, Alejandro Gutierrez, Daniel J. Goff, Ifat Geron, Anil Sadarangani, Christina A. M. Jamieson, Angela C. Court, Alice Y. Shih, Qingfei Jiang, Christina C. Wu, Kang Li, Kristen M. Smith, Leslie A. Crews, Neil W. Gibson, Ida Deichaite, Sheldon R. Morris, Ping Wei, Dennis A. Carson, A. Thomas Look, Catriona H. M. Jamieson

**Affiliations:** 1 Department of Medicine, Stem Cell Program and Moores Cancer Center, University of California San Diego, La Jolla, California, United States of America; 2 Department of Pediatric Oncology, Dana-Farber Cancer Institute and Children’s Hospital Boston, Boston, Massachusetts, United States of America; 3 Oncology Research Unit, Pfizer Global Research and Development, La Jolla Laboratories, San Diego, California, United States of America; National Cancer Institute, United States of America

## Abstract

**Background:**

Leukemia initiating cells (LIC) contribute to therapeutic resistance through acquisition of mutations in signaling pathways, such as NOTCH1, that promote self-renewal and survival within supportive niches. Activating mutations in NOTCH1 occur commonly in T cell acute lymphoblastic leukemia (T-ALL) and have been implicated in therapeutic resistance. However, the cell type and context specific consequences of NOTCH1 activation, its role in human LIC regeneration, and sensitivity to NOTCH1 inhibition in hematopoietic microenvironments had not been elucidated.

**Methodology and Principal Findings:**

We established humanized bioluminescent T-ALL LIC mouse models transplanted with pediatric T-ALL samples that were sequenced for NOTCH1 and other common T-ALL mutations. In this study, CD34^+^ cells from *NOTCH1^Mutated^* T-ALL samples had higher leukemic engraftment and serial transplantation capacity than *NOTCH1^Wild-type^* CD34^+^ cells in hematopoietic niches, suggesting that self-renewing LIC were enriched within the *NOTCH1^Mutated^* CD34^+^ fraction. Humanized NOTCH1 monoclonal antibody treatment reduced LIC survival and self-renewal in *NOTCH1^Mutated^* T-ALL LIC-engrafted mice and resulted in depletion of CD34^+^CD2^+^CD7^+^ cells that harbor serial transplantation capacity.

**Conclusions:**

These results reveal a functional hierarchy within the LIC population based on NOTCH1 activation, which renders LIC susceptible to targeted NOTCH1 inhibition and highlights the utility of NOTCH1 antibody targeting as a key component of malignant stem cell eradication strategies.

## Introduction

Seminal research suggests that leukemia relapse occurs because standard chemotherapy fails to eradicate self-renewing leukemia initiating cells (LIC) [1]–[15]. While human myeloid leukemia xenograft studies demonstrate that LIC reside at the apex of a cellular hierarchy and are capable of serially transplanting leukemia [1]–[3], [6], cellular subpopulations within diagnostic precursor B cell acute lymphoblastic leukemia (ALL) samples demonstrate greater functional and genetic heterogeneity [16], [17]. Recently, DNA copy number alteration (CNA) profiling coupled with xenograft analysis suggested that patients with BCR-ABL1 ALL harboring a predominant clone at diagnosis have increased rates of early relapse thereby linking LIC clonal dominance with a poorer prognosis [18].

In another leukemia subtype that is prone to early relapse [19], pediatric T cell acute lymphoblastic leukemia (T-ALL), serially transplantable LIC were found to be enriched in CD34^+^CD4^−^ and CD34^+^CD7^−^ fractions of newly diagnosed patient samples [12]. However, these results were obtained after suspension culture-mediated expansion prior to transplantation potentially leading to changes in LIC functional capacity. More recently, a CD7^+^CD1a^−^ glucocorticoid resistant LIC population, capable of engrafting leukemia in NOD/SCID IL2Rγnull (NSG) mice, was identified in primarily adult T-ALL without an *in vitro* expansion step [10]. While the LIC population was found to be an essential driver of therapeutic resistance and relapse, the NOTCH1 mutational status of the LIC population was not established; the cell surface phenotype changed during the prolonged engraftment period and niche dependence of LIC maintenance, which could ultimately contribute to relapse, was not elucidated. The high propensity for T-ALL relapse underscores the need for LIC characterization based on functional molecular drivers of survival and self-renewal and spatiotemporal tracking of niche dependence in bioluminescent serial xenotransplantation models. Together these compelling studies provided the impetus for investigating the potential LIC propagating capacity of NOTCH1 mutations, implicated in T-ALL therapeutic resistance [10] and sensitivity to targeted NOTCH1 inhibition within selective niches.

While T-ALL represents only 25% of adult and 15% of pediatric ALL cases, they share an increased risk of early systemic and isolated central nervous system relapse often in the setting of mutational NOTCH1 signaling pathway activation [20]. A recent series of studies showed that NOTCH activation is associated with improved early therapeutic response (reviewed in [21]). However, this early benefit translates into improved overall survival only in some series, most probably as a result of differences in therapy, and suggests that NOTCH-targeted therapies might represent promising therapeutic strategies. During normal hematopoiesis, NOTCH1 regulates cell fate decisions, proliferation and survival following ligand binding, which triggers a conformational change in the negative regulatory region (NRR) of the extracellular domain, enabling juxtamembrane ADAM protease cleavage [22], [23]. Subsequently, γ-secretase complex mediated intramembrane proteolysis releases an intracellular domain of NOTCH1 (ICN1), which translocates to the nucleus and activates transcription of NOTCH target genes [22], [24]. In T-ALL, somatic activating mutations in the NOTCH1 heterodimerization domain (HD) or PEST domain or alternatively loss-of-function mutations in FBXW7, a NOTCH1 E3 ubiquitin ligase, increase release or stability of ICN1. This, in turn, leads to transcriptional activation of genes that promote proliferation and survival such as MYC and HES1 [22], [24].

Despite a plethora of reports describing mechanisms of NOTCH1 activation in T-ALL, the cell type and context specific role of NOTCH1 activation in the maintenance of therapeutically resistant self-renewing human LIC has not been established. Thus, we sought to examine (1) whether molecularly characterized LIC can be identified among specific hematopoietic subpopulations in pediatric T-ALL without preceding *in vitro* culture, (2) the role of NOTCH1 activation in LIC propagation, and (3) whether LIC have an intrinsic predilection for specific hematopoietic niches. For these purposes, lentiviral luciferase-transduced CD34-enriched (CD34^+^) and CD34-depleted (CD34^-^) cells from molecularly characterized samples were transplanted into neonatal RAG2^−/−^γ_c_^−/−^ mice that permit high levels of human hematopoietic engraftment [6], [7]. In this study, the CD34^+^ fraction of pediatric *NOTCH1^Mutated^* T-ALL samples had enhanced survival and self-renewal potential, characteristic of LIC, compared with their CD34^+^ NOTCH1 wild-type (*NOTCH1^WT^*) counterparts. These *NOTCH1^Mutated^* LIC were uniquely susceptible to targeted inhibition with a therapeutic human NOTCH1 monoclonal antibody selective for the NRR (hN1 mAb), while normal hematopoietic progenitors were spared thereby highlighting the cell type and context specific effects of NOTCH signaling [13], and the importance of oncogenic addiction to NOTCH1 signaling in T-ALL LIC maintenance.

## Results

### T-ALL Molecular Characterization

Molecular characterization of CD34^+^ cells from 12 T-ALL patient samples was performed by targeted exon sequencing analysis and focused on genes commonly mutated in T-ALL, including NOTCH1, PTEN, PIK3R1 and FBXW7 (Table 1). Selective NOTCH1 DNA sequencing revealed activating mutations in six of eleven newly diagnosed pediatric T-ALL samples and in one relapsed young adult T-ALL sample (Table 1). In addition, CD34^+^ T-ALL cells derived from these 12 samples were further sequenced to identify PI3K, PTEN and FBXW7 pathway mutations common to pediatric T-ALL. Some cases harbored mutations in PTEN (patients 01, 05, 06, 11) or PIK3R1 (patient 05) genes (Table 1) [31]–[35]. These data demonstrate that mutations in NOTCH1 and other genes capable of promoting LIC survival co-exist in the CD34^+^ fraction of T-ALL samples.

**Table 1:** Patient characteristics.

T-ALL Code	Age	Diagnostic Immunophenotype	NOTCH1 HD Domain (exons 26,27)	NOTCH1 PEST Domain (exon 34)	PTEN (exon 1)	PIK3R1 (exons 12,13)	FBXW7 (exons 9,10)
01	4	CD45 (dim), TdT, CD2, CD3, CD5, CD7, CD4 (subset), CD8 (variable);CD34 (-), HLA-DR (-), CD33 (-), CD13 (-), CD19 (-), CD10 (-)	het c.4793GdelinsC/p.Arg1598Pro	wt	hom c.583–585 deletion/p.Phe195 deletion	N/A	wt
02*	2	CD45 (low), CD1, CD2, CD3, CD4, CD5, CD7, CD34; HLA-DR (-), CD33 (-),CD13 (-), CD10 (-), CD19 (-)	wt	wt	wt	wt	wt
03	16	CD45 (dim), TdT, sCD3 (subset), CD5, CD7, CD4 (subset), CD8, CD34(-),HLA-DR(-) and B lymphoid (-)	Homo p. Leu1574Pro; Homo p. Leu1574Pro	wt	wt	wt	N/A
04	17	CD45(dim), TdT, CD34 (bright), CD2, sCD3(dim), CD5(dim), CD7(dim),CD13/CD33 (dim). CD4 (-), CD8 (-), CD10 (-), CD17 (-), HLA-DR (-)	wt	wt	wt	wt	wt
05	6	CD45 (variable), TdT, CD34 (subset), CD2, sCD3 (variable), CD5, CD7, CD4 (variable), and CD8 (variable). CD10 (-), CD13 (-), CD33 (-), CD56 (-),CD117 (-) and HLA-DR (-)	Het p.Ala1552Thr	c.7541–7542delCT/p.Pro2515Arg (frame shift)	c.17_22del/p.Lys6_Glu7del	c.1739_1742delinsC/p.Tyr580_Leu581deli nsSer*	wt
06	7	CD45 (inter/dim),TdT, CD2, sCD3 (subset), CD4, CD5, CD7, CD8,CD10. HLA-DR(-), CD34(-), CD56(-)	wt	wt	compound deletion-insertion after bp 699/Arg233*	wt	wt
07	2	CD45 (dim), TdT, CD2, CD4, CD5, CD7 (bright), CD8. CD34 (-), HLA-DR (-),sCD3 (-), CD10 (-), CD13 (-), CD15 (-), CD33 (-), CD56 (-), CD117 (-)	wt	wt	wt	wt	wt
08	16	CD1, CD2, CD3, CD4, CD5, CD7, CD8, CD9, CD34, CD37, CD38, CD45.CD10 (-), CD13 (-), CD15 (-), CD19 (-), CD20 (-). HLA DR (-)	Hetc.4847_4848ins/p.1616Ile_(ProLeuArgHis)_1617Phe	wt	wt	wt	wt
09	10	N/A	wt	wt	wt	wt	wt
10	14	N/A	wt	wt	wt	wt	wt
11	19	CD45 (dim), TdT (dim), TCRγ/δ, CD1a, CD2, CD3, CD4, CD5, CD7,CD15, CD34	Het p. Ile1616_Phe1617insPLRH	wt	exon 7 frame shift	N/A	wt
12	23	CD45 (dim), CD2, CD5 (dim), CD7, CD13 (dim), CD34, CD52 (dim),TdT (dim)	het c.4587_4871del/p.Asn1529Lys (frame shift)	wt	wt	wt	wt

Diagnostic blast immunophenotypic analysis (in bulk patient samples) and DNA sequencing (in the CD34^+^ cell population) were performed for 12 pediatric T-ALL patient samples. Patient age range was 2–23 and mean age was 11.3 years. Diagnostic samples included patients 1–11 while sample 12 was donated by a patient with relapsed T-ALL. Diagnostic blast immunophenotypes were not available (N/A) for 2 of 12 patients. Treatment was performed according to protocol COG 05-01 for patients 1 to 7; protocol COG 9404 for patients 8 to 10; the Larson protocol for patient 11, and patient 12 received COG-ALL043 treatment. Targeted exon sequencing performed on T-ALL CD34^+^ LIC revealed NOTCH1 HD or PEST domain mutations in 6 of 12 T-ALL (patients 01, 03, 05, 08, 11, 12) samples. PTEN mutations and/or deletions were detected in 4 of 12 samples (patients 01, 05, 06, 11). Patient 5 also had a PIK3R1 mutation. ^#^ Indicates *NOTCH1^Mutated^* patient samples, and * indicates NOTCH1 activation in the absence of NOTCH1 mutation (*NOTCH1^High^*) by qRT-PCR.

### NOTCH1^Mutated^ T-ALL LIC are Serially Transplantable

**Figure 1:**
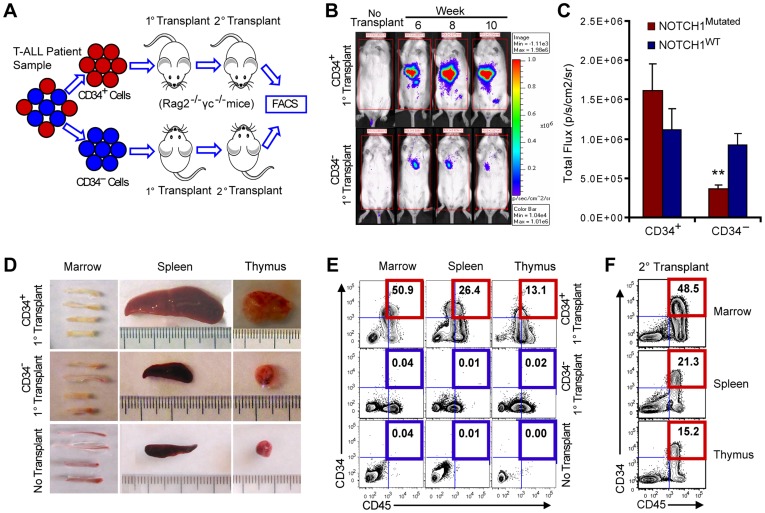
*NOTCH1^Mutated^* LIC serially transplant T-ALL. (A) Schema of T-ALL LIC mouse model. Equivalent numbers of human CD34^+^ and CD34^−^ cells derived from both NOTCH1 Mutated (NOTCH1^Mutated^ and NOTCH1 wild-type (NOTCH1^WT^) samples, as defined by DNA sequencing (Table 1), were selected using immunomagnetic beads from T-ALL blood or bone marrow, transduced with lentiviral luciferase and transplanted (50000 cells/mouse) intrahepatically into RAG2^−/−^γ_c_^−/−^ mice within 48 hours of birth. Engraftment was monitored over 10 weeks via non-invasive bioluminescent imaging system (IVIS 200, Caliper LifeSciences). After 10 weeks, mice were sacrificed and secondary transplants were performed with 50000 immunomagnetically-purified human CD34^+^ or CD45^+^ cells from primary CD34^+^ and CD34^−^ T-ALL engrafted mouse marrow, respectively. (B) T-ALL CD34^+^ cells from 12 of 12 T-ALL samples engrafted leukemia to varying extents in the marrow, spleen and thymus in all primary but only 10 of 11 serial transplant recipients. Representative bioluminescent images (captured on an IVIS 200 system) demonstrating engraftment of CD34^+^ (50000) NOTCH1^Mutated^ T-ALL (1B, upper) compared with an equivalent number of CD34^−^ cells (1B, lower). (C) Quantitative bioluminescent imaging (photons/second/cm^2^/sr) of CD34^+^ cell and CD34^−^ cell engraftment over 10 weeks following transplantation of NOTCH1^Mutated^ T-ALL samples (red, n = 4 patients) compared with NOTCH1^WT^ samples (blue, n = 4 patients). Mice (n = 76) transplanted with lentiviral luciferase-transduced NOTCH1^WT^ patient samples (Patients 04, 07, 09, 10) and mice (n = 79) transplanted with NOTCH1^Mutated^ patient samples (Patients 03, 05, 08, 11) were included in the bioluminescent imaging study (error bars, mean ± SEM. **, P = 0.0005, Student’s t-test). (D) Photographs depicting marrow, thymic and splenic size following NOTCH1^Mutated^ T-ALL CD34^+^ cell (upper panel) and CD34^−^ cell (middle panel) transplants compared with no transplant control mice (lower panel). (E) Representative FACS analysis demonstrating human CD45^+^ and CD34^+^ leukemic engraftment in the marrow, spleen and thymus in primary transplant recipients of NOTCH1^Mutated^ T-ALL Pt 05 CD34^+^ cells (CD34^+^1° transplant, upper panel) and CD34^−^ cells (CD34^−^ 1° transplant, middle panel) compared with no transplant control mice (lower panel). (F) Representative FACS analysis demonstrating human CD34^+^CD45^+^ serial leukemic engraftment in the marrow (upper), spleen (middle) and thymus (lower) following transplantation of secondary recipients (2° transplant) with 50000 CD34^+^ cells isolated from NOTCH1^Mutated^ T-ALL (Pt 05) primary recipients.

To determine if lentiviral luciferase-transduced CD34^+^ and CD34^−^ cells from *NOTCH1^Mutated^* and *NOTCH1^WT^* pediatric T-ALL samples differed in their capacity to propagate disease, quantitative non-invasive bioluminescent imaging was performed within 10 weeks of intrahepatic transplantation of neonatal RAG2^−/−^γ_c_^−/−^ mice (Figures 1A and B). Mice transplanted with CD34^+^ enriched *NOTCH1^Mutated^* T-ALL cells (patients 03, 05, 08, 11) demonstrated significantly greater leukemic engraftment than mice transplanted with CD34^−^ cells (Figures 1B and C; n = 79 mice, P = 0.0005, Student’s t-test). Conversely, both CD34^+^ and CD34^−^ fractions from *NOTCH1^WT^* T-ALL samples (patients 04, 07, 09, 10) exhibited equivalent engraftment capacity in primary transplant recipients (Figure 1C; n = 76 mice). Hence, CD34^+^ cells from *NOTCH1^Mutated^* samples gave rise to higher levels of bioluminescent engraftment in primary transplant recipients than their CD34^−^ counterparts, indicative of LIC enrichment in the CD34^+^ fraction in *NOTCH1^Mutated^* but not *NOTCH1^WT^* samples.

The predilection of *NOTCH1^Mutated^* T-ALL LIC for specific hematopoietic niches was determined in primary and serial transplants. Primary human *NOTCH1^Mutated^* T-ALL CD34^+^ LIC engraftment was typified by thymic (Figure 1D) and splenic (Figure 1D) enlargement as well as pale marrow due to replacement by leukemic cells (Figure 1D). Further analysis revealed that thymic (P<0.01; Student’s t test) and splenic (P<0.001, Student’s *t*-test) weights were significantly greater in both primary (1°) and secondary (2°) *NOTCH1^Mutated^* T-ALL LIC transplant recipients than in no transplant control mice (Figures S1A and B). Moreover, FACS analysis revealed robust CD34^+^ cell engraftment in marrow, spleen and thymus of primary and secondary *NOTCH1^Mutated^* T-ALL LIC transplanted mice (Figures 1E and F). CD45^−^ cells detected by FACS represent endogenous mouse cell populations present in the hematopoietic tissues. Notably, serially transplantable CD34^+^ cells from *NOTCH1^Mutated^* (patients 03, 05, 08, 11) and *NOTCH1^High^* (patient 02) T-ALL samples showed increased human *NOTCH1* (***, P<0.001; **, P<0.01) transcript levels compared to bone marrow engrafted with *NOTCH1^WT^* (patient 09) CD34^+^ T-ALL cells (Figure S1C). Taken together, these results suggest that NOTCH1-driven LIC have enhanced survival and self-renewal potential in supportive hematopoietic microenvironments.

### hN1 mAb Treatment Reduces NOTCH1^Mutated^ T-ALL LIC Survival

**Figure 2:**
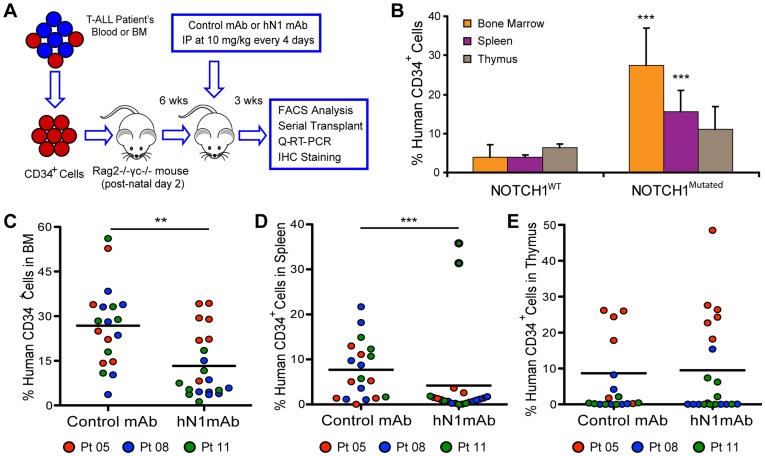
hN1 mAb treatment inhibits *NOTCH1^Mutated^* T-ALL LIC burden. (A) Schema of T-ALL LIC mouse model treatment with a selective anti-NOTCH1-NRR (hN1) mAb. Within 10 to 12 weeks of NOTCH1^Mutated^ T-ALL CD34^+^ (50000) cell transplantation into post-natal day 2 RAG2^−/−^γ_c_^−/−^ mice, intraperitoneal treatment was instituted for 3 weeks with a NOTCH1 (hN1, 10 mg/kg every 4 days) mAb or control mAb for 6 doses. Mice were sacrificed within one day of completion of dosing followed by further studies. (B) The average percentage of CD34^+^ engraftment was compared by FACS analysis of hematopoietic tissues (bone marrow, spleen and thymus) from mice that received transplants of T-ALL LIC from NOTCH1^Mutated^ patient samples (03, 05, 08, 11; n = 29 mice) and NOTCH1^WT^ patient samples (04, 06, 09, 10; n = 22 mice) (error bars ± SEM. ***, P<0.001, unequal variance Student’s t-test). (C) The average percentage of human CD34^+^ T-ALL LIC burden in the bone marrow (red, Patient 05, n = 6; blue, Patient 08, n = 7; green, Patient 11, n = 6) in control mAb treated mice (n = 19) and hN1 mAb treated mice (red, Patient 05, n = 7; blue, Patient 08, n = 7; green, Patient 11, n = 7) (**, P = 0.003 by Wilcoxon test) was compared by FACS analysis. Response in the bone marrow of mice transplanted with LIC from patients 05, 08 and 11 varied (Patient 05, P = 0.8308; Patient 08, P = 0.007; Patient 11, P = 0.017, by Wilcoxon test). In addition to NOTCH1 activating mutations, patient 05 harbored both PTEN and PI3KR1 mutations and patient 11 had a PTEN frameshift mutation, while patient 08 was wild-type at these loci (Table 1). (D) Average percentage of T-ALL CD34^+^ LIC burden in the mouse spleens following control mAb treatment (n = 19) compared to hN1 mAb treatment (n = 21) (***, P<0.001, Wilcoxon test). (E) Average percentage of T-ALL CD34^+^ LIC burden in the mouse thymus after control mAb treatment (n = 19) when compared to hN1 mAb treatment (n = 21) (no significant differences between the two groups). All results reflect data collected from two independent experiments.

The relative leukemic regenerative potential of *NOTCH1^Mutated^* (Patients 03, 05, 08, 11), and *NOTCH1^WT^* (Patients 04, 07, 09, 10) samples was determined in serial transplantation studies. FACS analysis of cells from bone marrow, spleen and thymus showed that while the levels of thymic engraftment were equivalent, *NOTCH1^Mutated^* T-ALL LIC gave rise to a significantly higher CD34^+^ leukemic burden in the marrow and spleen of primary transplant recipients than *NOTCH1^WT^* T-ALL samples (Figures 2A and B; ***, P<0.001; Student’s t test). Hence, we sought to determine whether (1) selective NOTCH1 inhibition could reduce LIC burden, (2) *NOTCH1^Mutated^* T-ALL LIC survival is dependent on activated NOTCH1 receptor signaling, and (3) selective NOTCH1 inhibition could spare *NOTCH1^WT^* or normal cord blood CD34^+^ progenitors in engrafted mice. For these purposes, *NOTCH1^Mutated^* T-ALL LIC-engrafted mice were treated with a selective NOTCH1-NRR/Fc mAb (hN1 mAb) that specifically inhibits NOTCH1 receptor signaling (Figures S2A and B).

Animals were treated with hN1 mAb (10 mg/kg) or a control mouse IgG1 mAb every 4 days for 3 weeks, and over this time period both antibodies were well-tolerated in treated animals (Figure S2C). As anticipated, treatment with the hN1 mAb had no detectable toxicity (e.g. intestinal) or deleterious effects on survival in mice, as this antibody does not bind to endogenous murine NOTCH1 and is expected to only target activity of human NOTCH1 in transplanted human cells. Following hN1 mAb treatment of *NOTCH1^Mutated^* T-ALL LIC-transplanted mice, FACS analysis revealed a significant reduction in leukemic CD34^+^ cell burden in both the marrow and spleen of hN1 mAb-treated mice (Figure 2C; **, P = 0.003 and Figure 2D, ***, P = 0.001, respectively, Wilcoxon test). Levels of CD34^+^ cell burden in the thymus were similar in both groups (Figure 2E), which is likely a result of relatively lower engraftment rates in this hematopoietic organ (Figure 2B). Notably, LIC from one T-ALL *NOTCH1^Mutated^* patient sample (patient 11), with a PTEN frame-shift mutation, retained sensitivity to hN1 inhibition (Figure 2). While survival of LIC from a sample (patient 05) with both PTEN and PIK3R1 mutations was not significantly inhibited in the bone marrow, LIC burden was significantly reduced in the spleen by hN1 mAb treatment (Figure 2C and D), highlighting the influence of additional mutations and microenvironmental context in responses to selective NOTCH1 inhibitory strategies. Although engraftment rates of normal human cord blood progenitors were low, the survival of normal CD34^+^ hematopoietic progenitors was not significantly reduced by targeted NOTCH1 inhibition (Figures S3A and B). These results suggest a greater functional dependence of *NOTCH1^Mutated^* T-ALL LIC on NOTCH1 signaling in selective hematopoietic niches compared to *NOTCH1^WT^* progenitors and normal hematopoietic stem cells (HSC).

**Figure 3:**
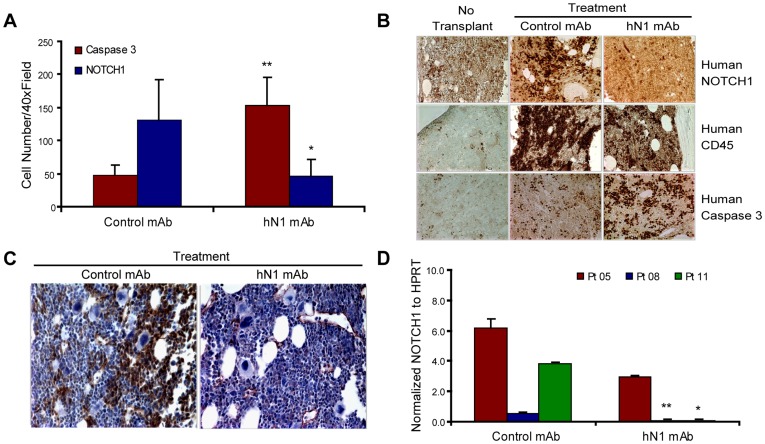
hN1 mAb treatment inhibits NOTCH1-driven LIC self-renewal. (A) Graph depicting average number of caspase 3 (red) and NOTCH1 (blue) positive cells (mean ± SEM), measured by marrow immunohistochemistry (cell number/randomized 40× fields) following hN1 versus control mAb treatment of T-ALL LIC (Patient 08) engrafted mice (**, P = 0.005, *, P = 0.046, respectively by two tailed Student’s t test with unequal variance). (B) Immunoperoxidase analysis of human NOTCH1, human CD45 and human Caspase 3 expression in no transplant control (left panel), compared with control mAb (middle panel) and hN1 mAb (right panel) treated NOTCH1^Mutated^ LIC (Patient 08) engrafted mice (40× magnification). (C) Following hN1 mAb treatment, immunoperoxidase staining of marrow sections was used to compare NOTCH1 intracellular domain (ICN1) expression (Patient 08) in control mAb (left) and hN1mAb (right) treated mice (40× magnification). (D) Quantitative RT-PCR assessment of relative reduction in HPRT normalized NOTCH1 transcript levels in human CD34^+^ cells derived from NOTCH1^Mutated^ (Patients 05, 08, 11) T-ALL LIC engrafted bone marrows following hN1 mAb or control mAb treatment (**, P<0.01; *, P<0.05, Student’s t-test). All results reflect data collected from two independent experiments.

Following hN1 mAb treatment, immunohistochemical analyses revealed a marked increase in levels of activated caspase 3, and a concomitant reduction in levels of NOTCH1 in *NOTCH1^Mutated^* (patient 08) T-ALL LIC-engrafted bone marrow compared with control IgG1 mAb-treated control bone marrow (Figures 3A and B; **, P = 0.005; *, P<0.05, Student’s t test). To assess whether hN1 mAb treatment could inhibit the generation of transcriptionally active NOTCH1 (intracellular NOTCH1, ICN1), which may be involved in promoting therapeutic resistance through induction of self-renewal, ICN1 immunohistochemical analysis was performed on bone marrow derived from the *NOTCH1^Mutated^* LIC-engrafted mice after treatment with hN1 mAb or IgG1 control mAb. Treatment with the hN1 mAb was associated with a reduction in bone marrow ICN1 levels (Figure 3C). These data corroborate that the antibody’s mechanism of action involves both interference with ligand binding (Figure S2B) and reduced human NOTCH1 cleavage and ICN1 generation (Figure 3C).

Strikingly, qRT-PCR analysis demonstrated a significant reduction in NOTCH1 expression in a subset of *NOTCH1^Mutated^* T-ALL samples (patients 08, 11) after treatment with hN1 mAb (Figure 3D, **, P<0.01; *, P<0.05). One *NOTCH1^Mutated^* sample (patient 05) showed only a trend towards reduction in NOTCH1 expression after hN1 mAb treatment. However, this patient harbored multiple additional mutations in other pathways (Table 1) that could contribute to resistance of the LIC population. Of note, one patient sample (patient 02) did not harbor a NOTCH1 mutation, as determined by DNA sequencing (Table 1), but exhibited increased NOTCH1 transcript levels (NOTCH1^High^) compared to cord blood progenitors (Figure S1C), as well as increased bone marrow serial transplantation potential (Figure S4). Similar to the *NOTCH1^Mutated^* samples, the CD34^+^ population of cells in the *NOTCH1^High^* sample was reduced following hN1 mAb treatment compared to control IgG.

Alternative NOTCH1 inhibitory strategies also recapitulated the effects of antibody-mediated NOTCH1 pathway down-regulation, as demonstrated by *in vitro* lentiviral NOTCH1 shRNA knock down experiments (Figure S5). NOTCH1-shRNA treatment of CD34^+^ cells derived from *NOTCH1^High^* (patient 02) or *NOTCH1^Mutated^* (patient 05) samples resulted in reduced expression levels of NOTCH1 mRNA (Figure S5A) and downstream target genes (c-MYC) (Figure S5B).

### hN1 mAb Treatment Inhibits NOTCH1-Driven LIC Self-renewal

**Figure 4:**
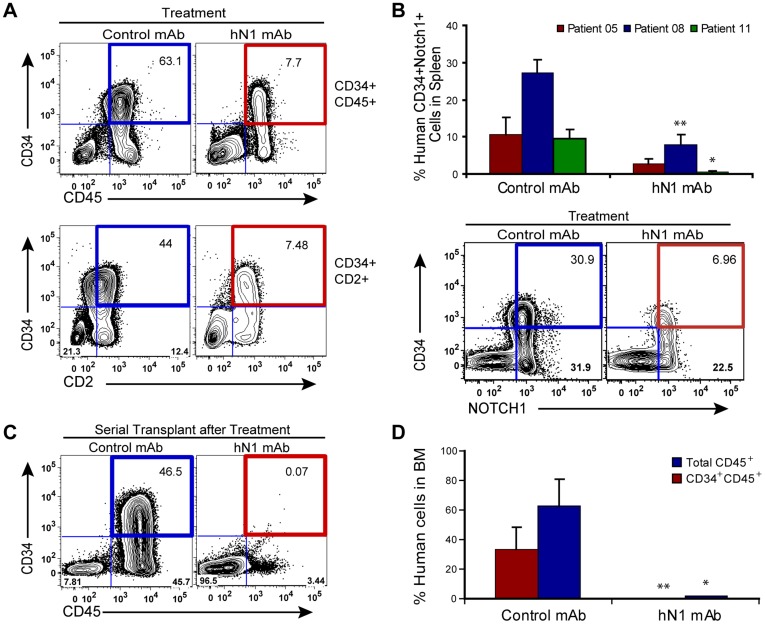
hN1 mAb treatment inhibits *NOTCH1^Mutated^* LIC burden. (A) Comparative FACS analysis of human CD34^+^CD45^+^ cells and CD34^+^CD2^+^ leukemic burden in the bone marrows from NOTCH1^Mutated^ LIC (Patient 11) engrafted mice following treatment with control mAb (left panel) or hN1 mAb (right panel). (B) FACS analysis of human CD34^+^ and NOTCH1^+^ cell survival in the mouse spleens following control mAb (n = 9) or hN1 mAb treatment (n = 9) of NOTCH1^Mutated^ LIC (Patients 05, 08, 11) engrafted mice (upper panel). Representative FACS plots show the reduction in both CD34^+^ and NOTCH1^+^ cell populations. (C) Representative FACS analysis demonstrating engraftment of CD34^+^CD45^+^ cells in the bone marrows of secondary (2°) transplant recipients following transplantation of control mAb (left panel) or hN1 mAb (right panel) treated bone marrow (Patient 11). (D) Graph of percent human T-ALL total CD45^+^ (blue) and CD34^+^CD45^+^ (red) cells in the bone marrows of 2° transplant recipients of control mAb (n = 6) and hN1 mAb (n = 6) treated NOTCH1-driven LIC (Patients 02, 11) (error bars ± SEM; P = 0.16, and P = 0.086, respectively, by Student’s t-test). All results reflect data collected from two independent experiments.

Following hN1 mAb treatment, FACS analysis revealed a reduction in both the populations of CD45^+^CD34^+^ cells (Figure 4A, upper panel) and immature T cells identified by CD34^+^CD2^+^ immunoreactivity (Figure 4A, lower panel) in *NOTCH1^Mutated^* LIC (Patient 11) engrafted mouse bone marrows, as well as a reduction of the CD34^+^NOTCH1^+^ cell population in bone marrow and spleen (Figure 4B). To determine whether hN1 mAb treatment inhibited NOTCH1-driven LIC self-renewal, human CD34^+^ cells selected from the bone marrows of hN1 mAb (n = 4) and IgG1 mAb (n = 3) treated mice were serially transplanted into untreated secondary (2°) recipients. After 12 weeks, FACS analysis showed that mice transplanted with human CD34^+^ T-ALL cells obtained from control IgG1 mAb-treated mice exhibited higher CD45^+^CD34^+^ leukemic burden compared to mice transplanted with CD34^+^ cells obtained from hN1 mAb-treated mice (Figure 4C). Conversely, hN1 mAb-treated human T-ALL LIC engrafted mice showed a significant reduction in both CD45^+^ cell burden (Figure 4D; **, P = 0.01, unequal variance two tailed Student’s t-test) and the CD45^+^CD34^+^ leukemic cell population (Figure 4D; *, P = 0.05) in the 2° transplant recipients, indicating that NOTCH1 inhibition abrogates T-ALL LIC self-renewal. Although *NOTCH1^Mutated^* T-ALL LIC demonstrated enhanced leukemic engraftment capacity compared with *NOTCH1^WT^* T-ALL CD34^+^ cells, the survival of *NOTCH1^Mutated^* T-ALL LIC appeared to be reliant on NOTCH1 signaling, thereby leading to enhanced sensitivity to hN1 mAb inhibition in a niche-dependent manner.

### Enrichment of LIC in the CD45^+^CD34^+^CD2^+^CD7^+^ Population and Depletion Following hN1 Antibody Treatment

**Figure 5:**
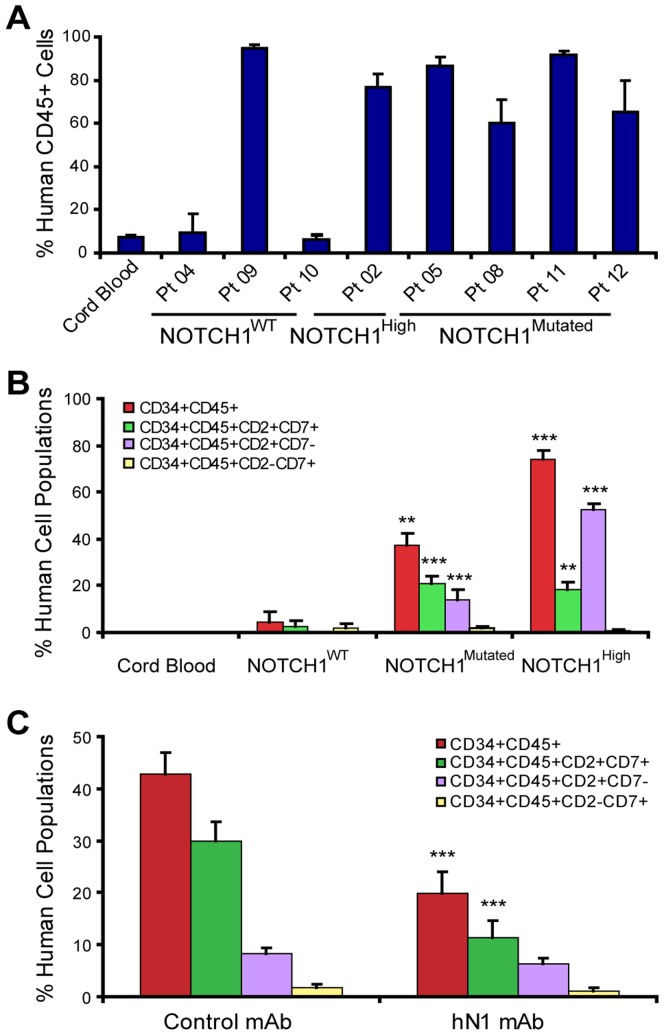
An expanded CD45^+^CD34^+^CD2^+^CD7^+^ population in *NOTCH1^Mutated^* T-ALL LIC is sensitive to hN1 mAb treatment. (A) Total human CD45^+^ cells including CD34^+^CD45^+^ and CD34^-^CD45^+^ in secondary (2°) transplant recipients were summarized by graphing the results of CD45 FACS analysis. Human cord blood CD34^+^ progenitors were used as a normal progenitor control, where the engraftment of human CD45^+^ cells in bone marrow was 7.13% ±1.3 (n = 6). (B) FACS analysis of pediatric T-ALL engrafted bone marrows revealed an expanded human CD45^+^CD34^+^CD2^+^CD7^+^ population in secondary (2°) transplant recipients that was more prominent in NOTCH1^Mutated^ T-ALL (Patient 05, n = 9; Patient 08, n = 8; Patient 11, n = 8; Patient 12, n = 3) and NOTCH1^High^ T-ALL (Patient 02, n = 10) transplanted mice than NOTCH1^WT^ T-ALL transplanted mice (Patient 09, n = 4; Patient 10, n = 3). Human cord blood CD34^+^ progenitors were used as a normal progenitor control (n = 6). The CD34^+^CD45^+^, CD34^+^CD45^+^CD2^+^CD7^+^, and CD34^+^CD45^+^CD2^+^CD7^−^ populations were significantly higher in the bone marrows of both NOTCH1^Mutated^ and NOTCH1^High^ T-ALL LIC transplanted mice (**, P<0.01; ***, P<0.001, Student’s t test) when compared with NOTCH1^WT^ T-ALL LIC transplanted mice. (C) FACS analysis of pediatric T-ALL LIC (Patient 05, n = 5 in control group, n = 6 in hN1 group; Patient 08, n = 5 in control group, n = 6 in hN1 group; Patient 11, n = 4 in control group, n = 5 in hN1 group) engrafted bone marrows showing a reduction in the human CD45^+^CD34^+^CD2^+^CD7^+^ cell population following hN1 mAb treatment compared to control IgG1 mAb (***, P<0.001, Student’s t test).

**Figure 6:**
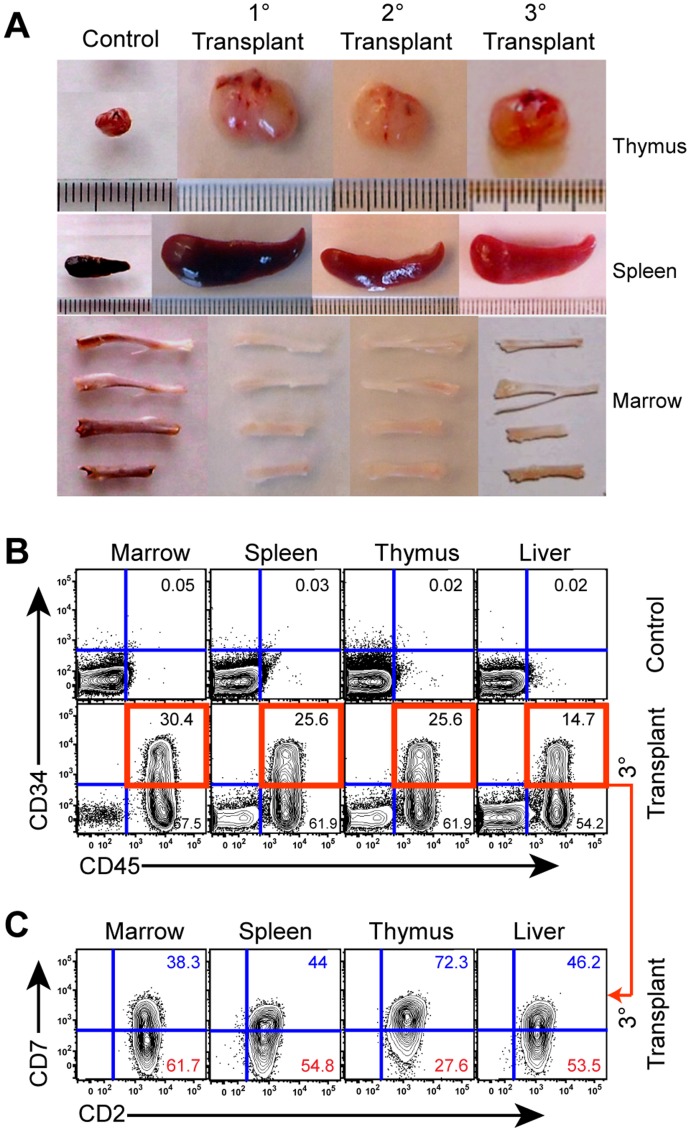
Leukemia regenerative capacity of the CD45^+^CD34^+^CD2^+^CD7^+^ population. (A) Representative photographs of hematopoietic organs following intrahepatic transplantation of 1 500 FACS purified CD34^+^CD2^+^CD7^+^Lin^−^ cells from a NOTCH1^Mutated^ T-ALL (Patient 05) sample demonstrates serial transplantation potential of this refined LIC population, as shown by the presence of an enlarged thymus, spleen and pale marrows over several transplantation generations. (B, C) FACS analysis of the tertiary (3°) transplant recipients of 30000 CD34^+^CD2^+^CD7^+^Lin^−^ cells sorted from a NOTCH1^Mutated^ T-ALL (Patient 11) revealed the persistence of an expanded human CD45^+^CD34^+^CD2^+^ (including CD7^+^ and CD7^−^) population in the transplanted mouse hematopoietic organs (bone marrow, spleen, thymus and liver).

Recent studies have highlighted the importance of CD7 expression in discriminating the LIC population in T-ALL [10], [36]. In this context, we hypothesized that early T cell markers such as CD7 and CD2 might be enriched, and these populations might be serially transplantable, in *NOTCH1*-driven T-ALL LIC xenografted mice. While overall engraftment was similar between *NOTCH1^High^* and *NOTCH1^Mutated^* transplanted samples (Figure 5A), FACS analyses revealed an expansion of CD45^+^CD34^+^CD2^+^CD7^+^ and CD45^+^CD34^+^CD2^+^CD7^−^ populations in *NOTCH1^Mutated^* (patients 05, 08, 11, 12) and *NOTCH1^High^* (patient 02) T-ALL samples when compared with *NOTCH1^WT^* (patients 04, 09, 10) T-ALL samples and cord blood (Figure 5B). Following serial transplantation of NOTCH1-activated LIC, FACS analyses revealed that the CD45^+^CD34^+^CD2^+^CD7^+^ population harbored serial leukemic transplantation potential at limiting doses (Table 2 and Figures 6A-C). To test our hypothesis that the CD2^+^CD7^+^ subset of the CD34^+^ human progenitor population identifies a LIC-enriched population in *NOTCH1^Mutated^* T-ALL samples, CD34^+^CD2^+^CD7^+^Lin^-^ cells from *NOTCH1^Mutated^* T-ALL samples (Patients 05 and 11) were FACS Aria purified and serial transplantations were performed. Serial transplantation of 1 500 CD34^+^CD2^+^CD7^+^Lin^−^ cells sorted from a *NOTCH1^Mutated^* T-ALL (Patient 05) sample resulted in marked thymic enlargement, splenomegaly and pale marrows indicative of robust leukemic engraftment (Table 2 and Figure 6A). Tertiary transplant experiments revealed that the human CD45^+^CD34^+^CD2^+^CD7^+^ population propagated leukemia and seeded hematopoietic niches, which was demonstrative of LIC self-renewal capacity (Figures 6B and C). As further evidence that this model recapitulates features of the human disease, infiltration of human CD45^+^ cells was detected in the brains of mice that received 3° transplants of the enriched LIC population (CD34^+^CD2^+^CD7^+^) from patient 11 (Figure S6).

**Table 2:** Niche-dependent LIC leukemic transplantation potential.

			1° Transplant Engraftment			2° Transplant Engraftment			3° Transplant Engraftment		
			CD34^+^ CD45^+^ Cells			CD34^+^ CD45^+^ Cells			CD34^+^ CD45^+^ Cells		
T-ALL LIC	T-ALL Code	Cell # Transplant (n)	BM	Spleen	Thymus	BM	Spleen	Thymus	BM	Spleen	Thymus
CD34^+^CD2^+^CD7^+^	05	12×10^3^ (n = 3)	19.9±1.0	7.7±0.9	3.3±0.6	6.7±0.3	0.6±0.1	37.8±3.1	N/A		
CD34^+^CD2^+^CD7^+^	05	1.5×10^3^ (n = 3)	70.1±2.8	37.4±9.6	50.2±6.6	7.1±1.3	2.7±0.9	5.8±2.7	2.5±1.4	3.5±2.8	0.5±0.1
CD34^+^CD2^+^CD7^−^	05	1.5×10^3^ (n = 3)	53.9±1.3	43.6±4.4	4.2±0.4	6.8±0.3	0.6±0.1	2.1±0.3	0.3±0.1	5.7±1.3	2.1±0.7
CD34^+^CD2^+^CD7^+^	11	30×10^3^ (n = 3)	N/A			11.7±6.6	9.3±3.3	2.3±1.2	10.9±8.1	11.3±5.9	11.1±4.1
CD34^+^CD2^+^CD7^−^	03	1.0×10^3^ (n = 1)	1.4±0.3	1.1±0.6	0.4±0.2	2.5±1.4	5.0±2.8	0.5±0.1	N/A		

1000, 1500, 12000, and 30000 CD34^+^CD2^+^CD7^+^Lin^-^ and CD34^+^CD2^+^CD7^-^Lin^-^ cells were sorted from T-ALL patient samples (Patients 03, 05, 11) and transplanted into neonatal immune deficient (RAG2^−/−^γ_c_^−/−^) mice. Numbers of cells transplanted and numbers of mice for each primary (1°) transplant experiment are indicated in the table, and results are reported as mean percentages ± SEM. Serial transplantations were performed using 50000 bone marrow cells derived from the engrafted mice. For secondary (2°) transplants, five experiments were performed with an average of 4.0±0.32 mice transplanted per experiment. For tertiary (3°) transplants, three experiments were performed with an average of 4.0±0.58 mice transplanted per experiment.

We then sought to determine whether this LIC-enriched population was sensitive to hN1 antibody treatment. Remarkably, compared to control mAb-treated animals transplanted with the bulk CD34^+^ population, in hN1 mAb-treated T-ALL LIC engrafted mice, there was a significant reduction in the CD34^+^CD2^+^CD7^+^ population, but not the CD34^+^CD2^+^CD7^−^ population (Figure 5C). Taken together, in *NOTCH1^Mutated^* T-ALL samples, a CD45^+^CD34^+^CD2^+^CD7^+^ population is enriched for LIC that demonstrate serial leukemic transplantation capacity, and these cells are selectively targeted and depleted by hN1 antibody therapy.

## Discussion

Cumulative reports reveal the protean nature of NOTCH signaling in the maintenance of normal and malignant hematopoiesis [13], [24]–[27], [37], [38]. While Notch2 signaling regulates regeneration of mouse long-term HSC, ligand-driven NOTCH1 activation induces human hematopoietic progenitor expansion and differentiation [28], [39]. Ligand binding to the NOTCH1 extracellular domain activates ADAM family metalloprotease and γ-secretase complex-mediated cleavage and intracellular release of the NOTCH1 intracellular domain (ICN1). Subsequently, nuclear translocation of ICN1 followed by engagement of transcriptional activators such as CBF1/Su(H)/Lag2 (CSL) and mastermind-like (MAML) sets the stage for NOTCH1 target gene transcription. Conversely, activation of NOTCH1 signaling through gain-of-function mutations in *NOTCH1*, first described in T-ALL [24], or loss-of-function mutations in *NOTCH1* regulators, such as *FBXW7* and *NUMB*, has been linked to therapeutic recalcitrance of hematologic malignancies [40], [41]. Chronic antagonism of both NOTCH1 and NOTCH2 processing with small molecule inhibitors of the γ-secretase complex has been associated with loss of intestinal crypt progenitor cells, thereby providing the impetus for development of selective NOTCH1 inhibitors [42]. Recent pre-clinical studies demonstrate that inhibition of NOTCH1 using synthetic stapled peptides or monoclonal antibody-mediated strategies effectively decreases T-ALL cell line growth [22], [23]. However, the consequences of selective NOTCH1 inhibition for normal hematopoietic progenitor and patient-derived T-ALL LIC survival and self-renewal have been unclear.

In this study, CD34^+^ cells from 6 of 12 T-ALL samples harbored NOTCH1 activating mutations. In these patients, *NOTCH1^Mutated^* CD34^+^ LIC had greater engraftment and serial transplantation potential than their CD34^−^ counterparts. Conversely, both CD34^+^ and CD34^−^ subpopulations from *NOTCH1^WT^* samples harbored roughly equivalent bioluminescent engraftment potential, albeit at lower levels than *NOTCH1^Mutated^* LIC and with lower serial transplantation capacity. With the exception of one sample (patient 02) that harbored high NOTCH1 transcript levels in the absence of identifiable *NOTCH1* mutations, bioluminescent imaging and FACS analyses of leukemic engraftment suggest that phenotypic markers other than CD34 will be needed to identify LIC in the *NOTCH1^WT^* samples. In contrast to experiments with *NOTCH1^WT^* and normal cord blood CD34^+^ samples, *NOTCH1^Mutated^* LIC survival was significantly impaired by selective hN1 mAb-mediated inhibition, concomitant with reductions in ICN1 and *NOTCH1* mRNA expression and protein levels. Furthermore, serial transplantation potential was also reduced by hN1 mAb treatment of mice transplanted with NOTCH1-activated T-ALL samples. Thus, *NOTCH1^Mutated^* CD34^+^ cells from these pediatric T-ALL patients constitute the apex of a leukemic hierarchy.

Notably, patient samples with NOTCH1 activation, conferred either by mutation or elevated WT NOTCH1 expression levels, show enrichment of a subset of the CD34^+^ human progenitor cell population distinguished by co-expression of CD2 and CD7. Seminal studies reveal that CD7 expression enriches for a therapeutically recalcitrant LIC population [10], [36]. Our analyses of the serial transplantation capacity of the CD34^+^CD2^+^CD7^+^ population reveal that this population is maintained over multiple generations of T-ALL LIC transplantation, and these cells harbor robust leukemic initiating potential in medullary and extramedullary reservoirs of resistance. In experiments aimed at elucidating the fate of these cells in mice treated with hN1 mAb, we observed a significant reduction in this population compared to animals that received control IgG1 antibody. Taken together, these data further refine the markers that identify LIC in *NOTCH1^Mutated^* T-ALL samples, and demonstrate that the CD34^+^CD2^+^CD7^+^ population is sensitive to and depleted following hN1 mAb treatment. While in the present studies, our analyses of the refined LIC marker were focused on the *NOTCH1^Mutated^* samples, additional markers, or activation of other receptor-mediated signaling pathways such as insulin-like growth factor 1 receptor [43], may also be informative to determine the leukemic potential of LIC in non-*NOTCH1^Mutated^* T-ALL patients.

While mutations in tumor suppressor genes co-exist in some samples, *NOTCH1^Mutated^* T-ALL LIC appear to be oncogenically addicted to NOTCH1 activation, rendering them uniquely susceptible to inhibition with a NOTCH1-targeted mAb, hN1. In contrast, hN1 mAb treatment did not significantly impair the survival of normal human hematopoietic progenitor cells. This favorable therapeutic index may be explained, at least in part, by mouse models of hematopoiesis, which demonstrate that Notch2, rather than Notch1, regulates mouse HSC regeneration [30]. In summary, characterization of LIC based on functional molecular drivers provides a useful paradigm for identification and selective elimination of malignant stem cells. Moreover, these findings provide a compelling rationale for clinical evaluation of hN1 mAb therapy in clinical trials aimed at eliminating self-renewing LIC that promote therapeutic resistance and relapse in T-ALL and potentially in other NOTCH1-driven malignancies.

## Materials and Methods

### Ethics Statement

Dr. Jamieson is the PI on an existing Institutional Review Board (IRB) approval for tissue banking, entitled “Protocol 070678: Permission to Collect Blood and/or Bone Specimens and/or Tumor Samples and/or Saliva from Patients with Hematology Problems for Research (Adult).” Approval was obtained from the UCSD Human Research Protections Program. Consent is always written, and clinical investigations were conducted according to the principles expressed in the Declaration of Helsinki. The Human Research Protections Program office assists researchers in complying with federal, state and University policies regarding experimentation involving human subjects, and oversees the review and conduct of research conducted by federally registered IRBs. This study was carried out in strict accordance with the recommendations of the Institutional Animal Care and Use Committee (IACUC) at the University of California, San Diego. The protocol was approved by the Committee under Animal Use Protocol Number S06015. All efforts were made to minimize suffering.

### Subjects and Samples

T-ALL patient samples were obtained from Moores Cancer Center, University of California San Diego and Dana-Farber Cancer Institute, Harvard Medical Schools. Patient consent forms were required from all patients or their legal guardians (if minors) for all samples collected for the study. At each collection site, samples were obtained under Institutional Review Board (IRB) approval. Peripheral blood mononuclear cells (PBMC) were purified by Ficoll-Hypaque centrifugation prior to fluorescence-activated cell sorting (FACS) analysis and viable cryopreservation in liquid nitrogen. Patient age range was 2–23 and mean age was 11.3 years. Diagnostic samples included patients 1–11, while the sample 12 was donated by a patient with relapsed T-ALL. Diagnostic blast immunophenotypes were not available (N/A) for 2 of 12 patients. Treatment was performed according to protocol COG 05-01 for 01 to 07; protocol COG 9404 for patients 8 to 10; the Larson protocol for patient 11 and patient 12 received COG-ALL043 treatment.

### Bioluminescent Humanized T-ALL LIC Models

Methods required for establishment of bioluminescent LIC models using lentivirus-luciferase transduced primary patient samples in neonatal RAG2^−/−^γ_c_^−/−^ mice were described previously [6]. All animal experiments were approved by the Animal Experimental Committee of the University of California San Diego and were performed according to NIH recommendations for animal use.

### Human CD34 Initiating Cell Isolation and Immunophenotypic Analysis

Immunophenotypic analysis was performed on all samples (FACSAria II system, BD Biosciences, Franklin Lakes, NJ). Human CD34^+^ cells were purified from T-ALL peripheral blood using a CD34 MicroBead Kit (Miltenyi Biotec, Auburn, CA) and CD34^+^ cell purity was assessed by FACS. For FACS sorting, mouse IgG1s conjugated to PE, FITC, or APC were used as isotype controls (BD Biosciences). Human CD45, CD34, CD38, CD2, and CD7 expression was assessed using anti-CD45-V450, anti-CD34-APC, anti-CD38-PE-Cy7, anti-CD2-FITC and anti-CD7-PE, respectively, together with a lineage cocktail including PE-Cy5.5-conjugated antibodies against human CD4, CD8, CD14, CD19 and CD56. All antibodies for cell sorting FACS analysis were obtained from BD Biosciences. Propidium iodide (PI) was used to exclude dead cells and at least 10000 events were acquired for each sample.

### Lentiviral Transduction and Transplantation

Approximately 50000 human CD34^+^ and CD34^−^ cells derived from both NOTCH1^Mutated^ and NOTCH1^WT^ samples (defined by DNA sequencing) were selected from T-ALL blood or bone marrow with the aid of immunomagnetic beads or FACSAria, and transduced with lentiviral luciferase which effectively transduced an average of 15–25% of cells, as measured by FACS (data not shown). Cells were transplanted intrahepatically into RAG2^−/−^γ_c_^−/−^ mice within 48 hours of birth [6]. For NOTCH1 shRNA knock down experiments, 100000 CD34^+^ cells selected from the T-ALL patient samples and cord blood (All Cells, Emeryville, CA) were transduced with lentiviral vectors expressing shRNA targeting human NOTCH1 (Dharmacon, Lafayette, CO).

### Establishment, Treatment and Analysis of LIC Mouse Model

Humanized LIC and normal hematopoietic progenitor mouse models were established by intrahepatic transplantation of neonatal RAG2^−/−^γ_c_^−/−^ mice with lentiviral luciferase-transduced CD34^+^ progenitor cells from 12 T-ALL samples or normal cord blood samples. Engraftment was monitored with the aid of an IVIS 200 system (Caliper Life Sciences, Inc.). LIC mouse models were dosed starting at 6 weeks of age with either NOTCH1 mAb or IgG1 mAb control (both provided by Pfizer, Inc., La Jolla, CA) at 10 mg/kg intraperitoneally every 4 days for an average of 6 doses. To test whether hN1 mAb treatment could eliminate LIC in humanized T-ALL mouse models, mice were sacrificed one day after the last dose. At this endpoint (approximately 10 weeks of age) the majority of animals were sacrificed before the disease was evident. Bone marrow, spleen, thymus and liver were collected and processed for FACS analysis of human CD45, CD34, CD2, CD7 and NOTCH1 expression. NOTCH1 FACS analysis was performed using the same mAb as was used for treatment. Immunohistochemistry was performed using antibodies against NOTCH1 (1∶50 dilution, Pfizer, Inc.), CD45 (1∶100 dilution, Cell Signaling, Danvers, MA), activated caspase 3 (1∶50 dilution, Abcam, Cambridge, MA), as well as NOTCH1 intracellular domain (1∶50 dilution, ICN1, Epitomics, Burlingame, CA) to examine expression levels in bone marrow sections following treatment with either control IgG1 mAb or hN1 mAb.

### Statistical Analysis

All statistical tests were performed for two sided p values. Continuous variables for each comparison group were assessed for distribution through univariate statistics. If the assumption of normal distribution could be supported, then the Student’s t-test was performed for comparison of two samples with assessment of equality of variance with an F statistic. If the assumption of normal distribution was not supported, nonparametric testing was performed with the two sample Wilcoxon test using the t approximation for samples with N of less than 20.

## Supporting Information

Figure S1 **Self-renewing LIC activate NOTCH1.** (A) Graph of mean thymic weight (grams) in primary (1°, n = 6) and secondary (2°, n = 8) *NOTCH1^Mutated^* T-ALL LIC (CD34^+^ cells) transplant recipients compared with no transplant control mice (n = 4) (error bars ± SEM. **, P<0.01, unequal variance two tailed Student’s t test). (B) Graph of mean splenic weight (grams) in 1° (n = 6) and 2° (n = 8) T-ALL LIC (CD34^+^ cells) transplant recipients compared with no transplant control mice (n = 4) (mean ± SEM. ***, P<0.001, unequal variance two sided Student’s t test). (C) Normalized NOTCH1 transcript levels to HPRT in engrafted human CD34^+^ cells following transplantation of *NOTCH1^Mutated^* T-ALL (Patients 03, 05, 08, 11), NOTCH1^High^ T-ALL (Patient 02), and *NOTCH1^WT^* T-ALL (Patient 09) samples compared with normal human cord blood CD34^+^ cells (**, P<0.01; ***, P<0.001; unequal variance two tailed Student’s t test). This experiment was repeated 3 times.
(PDF)

Figure S2 **Anti Notch1-NRR mAb specifically inhibits NOTCH1 receptor signaling and is well-tolerated in the humanized T-ALL LIC mouse model.** (A) Human NOTCH1-negative regulatory region/Fc (N1-NRR) or NOTCH2-NRR/Fc fusion protein (N2-NRR) expression plasmids were transiently transfected into Freestyle 293F cells (Invitrogen). The supernatants were coated in 96-well ELISA plates at 10 mg/mL. Purified hN1 mAb was added to the wells at the indicated concentrations. Graph of mean O.D. 450 value in ELISA binding assays demonstrates control mAb (blue) and anti-NOTCH1-NRR (hN1, red) mAb specificity for human NOTCH1 receptor versus NOTCH2 receptor. (B) NOTCH1 luciferase (NOTCH1+Luc) reporter assays utilized DLL4-coated plates. Graph depicts (blue bars) mean luciferase activity in BSA, DLL4, DLL4+control mAb and DLL4+ hN1 mAb (20 µg/mL) treated wells (mean ± SEM). (C) Mouse weight monitoring during dosing time (from day 1 to day 21) demonstrates that hN1 mAb treatment was well-tolerated in the engrafted mice (Control group, n = 24; hN1 mAb group, n = 28 mice). No significant weight loss was detected throughout the 3-week dosing period.
(PDF)

Figure S3 **hN1 mAb treatment spares normal hematopoietic progenitors.** (A) Representative FACS plots of human CD34 and CD45 bone marrow engraftment in mice (n = 6) transplanted with 50 000 normal human CD34^+^ cord blood cells from 3 different donors and treated with control mAb or hN1 mAb. (B) Graph of percent human CD45^+^ (blue) cells (error bars ± SEM; P = 0.16) and human CD34^+^CD45^+^ (red) cells (error bars ± SEM; P = 0.21) surviving in bone marrows of human cord blood CD34^+^ cells (from 3 different donors) transplanted mice following intraperitoneal treatment with control mAb (10 mg/kg, n = 6) or hN1 mAb (10 mg/kg, n = 6) every 4 days for 3 weeks.
(PDF)

Figure S4 **hN1 mAb treatment inhibits NOTCH1^High^ LIC self-renewal.** (A) Graph of HPRT-normalized q-RT-PCR results showing NOTCH1 transcript levels (blue bars) in normal cord blood CD34^+^ cells compared with engrafted T-ALL CD34^+^ cells from serially transplanted LIC from a NOTCH1^High^ patient sample (Patient 02). This experiment was repeated 3 times. (B) Graph of percent human CD34^+^ cell engraftment determined by FACS analysis in marrow, spleen and thymus of secondary (2°) transplant recipients of T-ALL NOTCH1^High^ LIC (Patient 02, n = 10). (C) Representative photographs depicting characteristics of serially transplanted mouse bone marrows derived from control mAb and hN1 mAb treated NOTCH1^High^ LIC engrafted mice (Patient 02). (D) FACS analysis demonstrating CD34^+^CD45^+^ LIC engraftment in bone marrow following serial transplantation of control mAb and hN1 mAb treated NOTCH1^High^ LIC (Patient 02).
(PDF)

Figure S5 **Human** ***NOTCH1*** **shRNA inhibits NOTCH1 and downstream genes in T-ALL patient samples.** (A) Normalized NOTCH1 expression to HPRT in CD34^+^ cells derived from T-ALL patient samples (patient 02 and 05) after treatment with *NOTCH1*-targeting shRNA expressed in a lentiviral vector with an MOI of 90 (error bars ± SEM, **, P<0.001 by Student’s t-test). (B) HPRT-Normalized c-MYC expression (error bars ± SEM; *, P<0.01 by Student’s t-test). These experiments were repeated 3 times.
(PDF)

Figure S6 **Human cell infiltration in the tertiary CD34^+^CD2^+^CD7^+^ cells transplanted mouse brain.** 30 000 CD34^+^CD38^+^CD2^+^CD7^+^Lin^−^ cells sorted from T-ALL patient 11 with the aid of FACS were intrahepatically transplanted into neonatal RAG2^−/−^g_c_^−/−^ mice, and serial transplantation were done by 50 000 mouse BM cells. Tertiary transplanted mouse brain was fixed in 4% PFA and 30% sucrose overnight, separately, and then embedded in OCT for section with the thickness of 16 µm. Mouse brain sections were stained with anti-human CD45 antibody, mounted with prolong gold (Invitrogen). Images were taken under the Fluoview FVi10 confocal microscope (Olympus). (A) H & E staining of the mouse ventricular area. Scale bar is 100 µm. (B) Human CD45 Immunostaining of no transplant control mouse brain. DAPI detects the mouse cell nuclei. Scale bar is 20 µm. (C) Human CD45 Immunostaining of 3° transplant mouse brain (patient 11). Pink cell surface staining represents the human CD45, and the blue staining is the cell nuclei. Scale bar is 20 µm.
(PDF)
