# Supplementary figures and images for: NOTCH1 Signaling Promotes Human T-Cell Acute Lymphoblastic Leukemia Initiating Cell Regeneration in Supportive Niches
Source: PLoS One. 2012 Jun 29;7(6):e39725. doi: 10.1371/journal.pone.0039725 (PMC3387267; doi:10.1371/journal.pone.0039725)

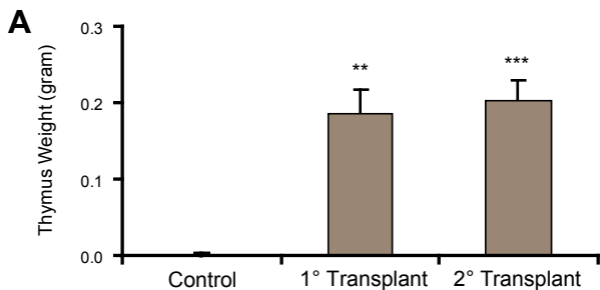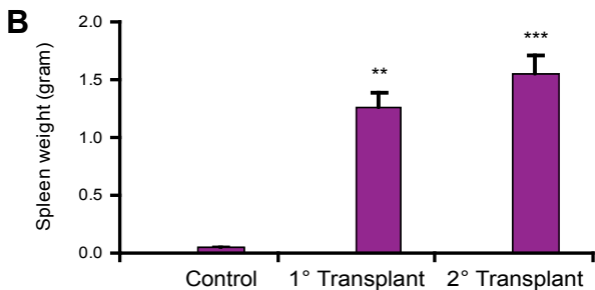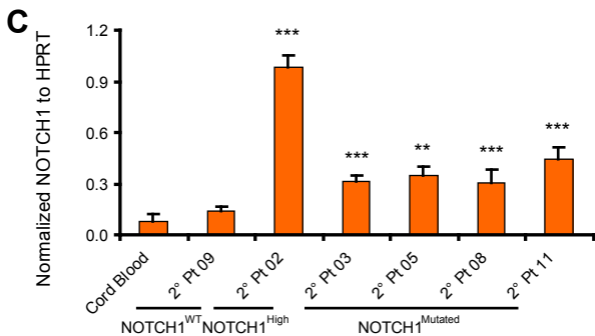

Supplement: Figure S1 — Self-renewing LIC activate NOTCH1. (A) Graph of mean thymic weight (grams) in primary (1°, n = 6) and secondary (2°, n = 8) NOTCH1Mutated T-ALL LIC (CD34+ cells) transplant recipients compared with no transplant control mice (n = 4) (error bars ± SEM. **, P<0.01, unequal variance two tailed Student’s t test). (B) Graph of mean splenic weight (grams) in 1° (n = 6) and 2° (n = 8) T-ALL LIC (CD34+ cells) transplant recipients compared with no transplant control mice (n = 4) (mean ± SEM. ***, P<0.001, unequal variance two sided Student’s t test). (C) Normalized NOTCH1 transcript levels to HPRT in engrafted human CD34+ cells following transplantation of NOTCH1Mutated T-ALL (Patients 03, 05, 08, 11), NOTCH1High T-ALL (Patient 02), and NOTCH1WT T-ALL (Patient 09) samples compared with normal human cord blood CD34+ cells (**, P<0.01; ***, P<0.001; unequal variance two tailed Student’s t test). This experiment was repeated 3 times. (PDF) [file pone.0039725.s001.pdf]

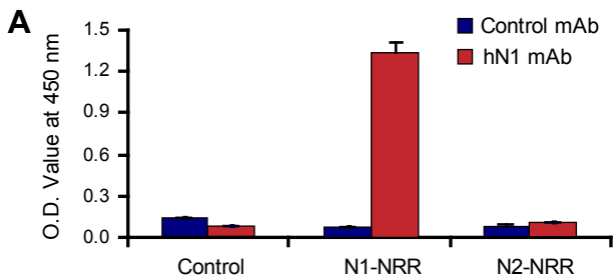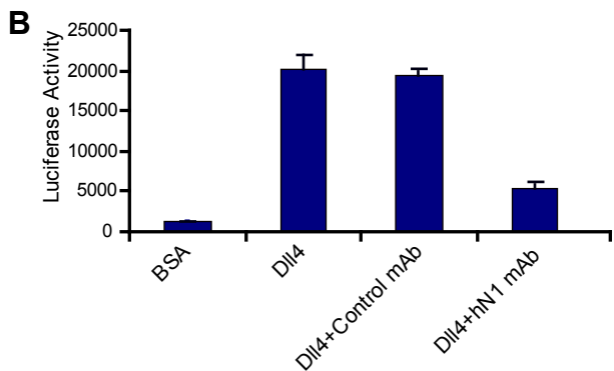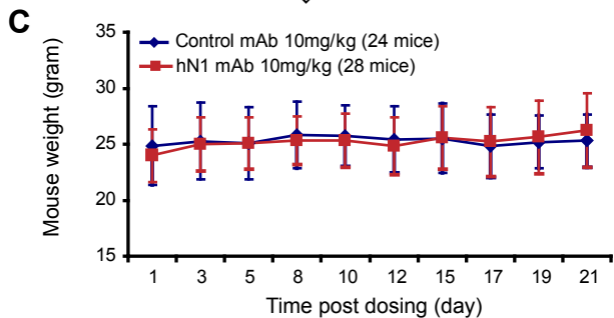

Supplement: Figure S2 — Anti Notch1-NRR mAb specifically inhibits NOTCH1 receptor signaling and is well-tolerated in the humanized T-ALL LIC mouse model. (A) Human NOTCH1-negative regulatory region/Fc (N1-NRR) or NOTCH2-NRR/Fc fusion protein (N2-NRR) expression plasmids were transiently transfected into Freestyle 293F cells (Invitrogen). The supernatants were coated in 96-well ELISA plates at 10 mg/mL. Purified hN1 mAb was added to the wells at the indicated concentrations. Graph of mean O.D. 450 value in ELISA binding assays demonstrates control mAb (blue) and anti-NOTCH1-NRR (hN1, red) mAb specificity for human NOTCH1 receptor versus NOTCH2 receptor. (B) NOTCH1 luciferase (NOTCH1+Luc) reporter assays utilized DLL4-coated plates. Graph depicts (blue bars) mean luciferase activity in BSA, DLL4, DLL4+control mAb and DLL4+ hN1 mAb (20 µg/mL) treated wells (mean ± SEM). (C) Mouse weight monitoring during dosing time (from day 1 to day 21) demonstrates that hN1 mAb treatment was well-tolerated in the engrafted mice (Control group, n = 24; hN1 mAb group, n = 28 mice). No significant weight loss was detected throughout the 3-week dosing period. (PDF) [file pone.0039725.s002.pdf]

# A

## Normal CD34<sup>+</sup> Cell Transplant

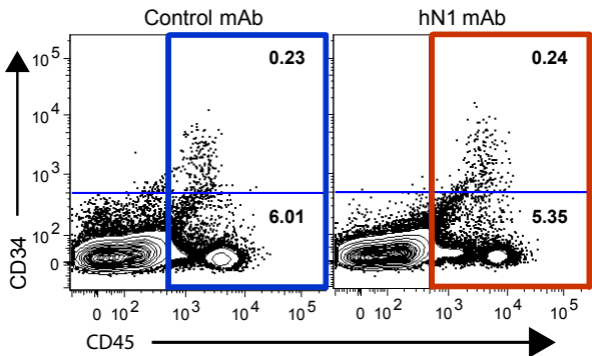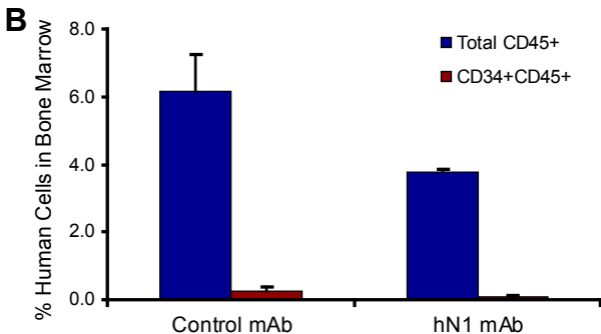

Supplement: Figure S3 — hN1 mAb treatment spares normal hematopoietic progenitors. (A) Representative FACS plots of human CD34 and CD45 bone marrow engraftment in mice (n = 6) transplanted with 50 000 normal human CD34+ cord blood cells from 3 different donors and treated with control mAb or hN1 mAb. (B) Graph of percent human CD45+ (blue) cells (error bars ± SEM; P = 0.16) and human CD34+CD45+ (red) cells (error bars ± SEM; P = 0.21) surviving in bone marrows of human cord blood CD34+ cells (from 3 different donors) transplanted mice following intraperitoneal treatment with control mAb (10 mg/kg, n = 6) or hN1 mAb (10 mg/kg, n = 6) every 4 days for 3 weeks. (PDF) [file pone.0039725.s003.pdf]

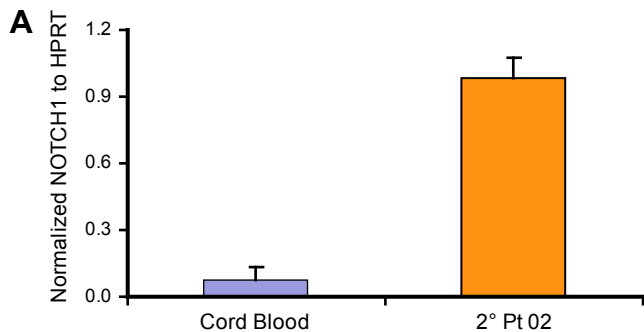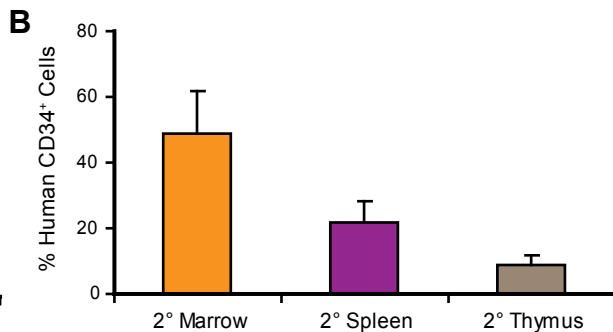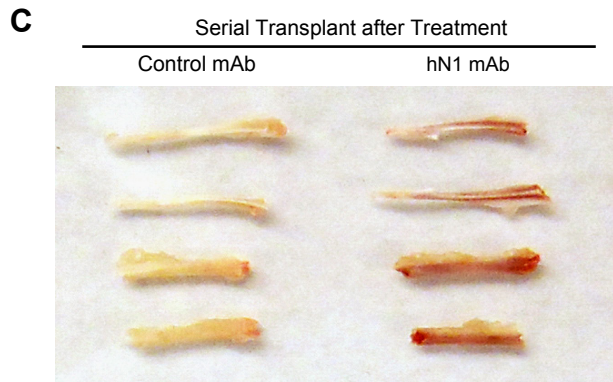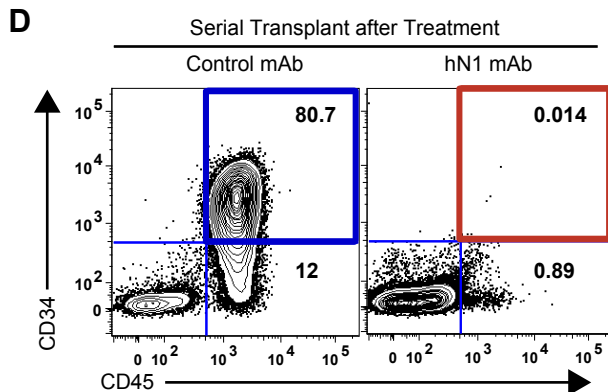

Supplement: Figure S4 — hN1 mAb treatment inhibits NOTCH1High LIC self-renewal. (A) Graph of HPRT-normalized q-RT-PCR results showing NOTCH1 transcript levels (blue bars) in normal cord blood CD34+ cells compared with engrafted T-ALL CD34+ cells from serially transplanted LIC from a NOTCH1High patient sample (Patient 02). This experiment was repeated 3 times. (B) Graph of percent human CD34+ cell engraftment determined by FACS analysis in marrow, spleen and thymus of secondary (2°) transplant recipients of T-ALL NOTCH1High LIC (Patient 02, n = 10). (C) Representative photographs depicting characteristics of serially transplanted mouse bone marrows derived from control mAb and hN1 mAb treated NOTCH1High LIC engrafted mice (Patient 02). (D) FACS analysis demonstrating CD34+CD45+ LIC engraftment in bone marrow following serial transplantation of control mAb and hN1 mAb treated NOTCH1High LIC (Patient 02). (PDF) [file pone.0039725.s004.pdf]

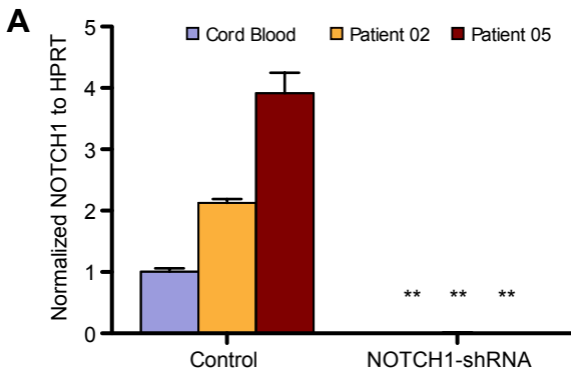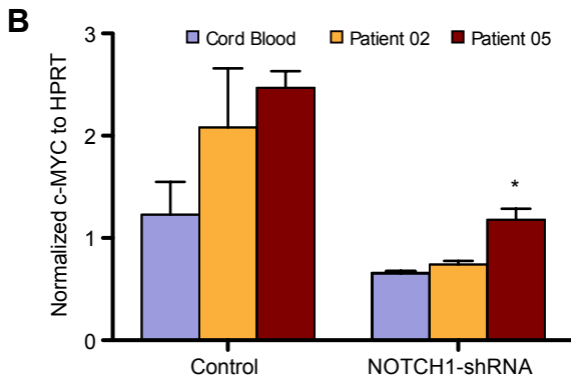

Supplement: Figure S5 — Human NOTCH1 shRNA inhibits NOTCH1 and downstream genes in T-ALL patient samples. (A) Normalized NOTCH1 expression to HPRT in CD34+ cells derived from T-ALL patient samples (patient 02 and 05) after treatment with NOTCH1-targeting shRNA expressed in a lentiviral vector with an MOI of 90 (error bars ± SEM, **, P<0.001 by Student’s t-test). (B) HPRT-Normalized c-MYC expression (error bars ± SEM; *, P<0.01 by Student’s t-test). These experiments were repeated 3 times. (PDF) [file pone.0039725.s005.pdf]

**A** H & E Staining

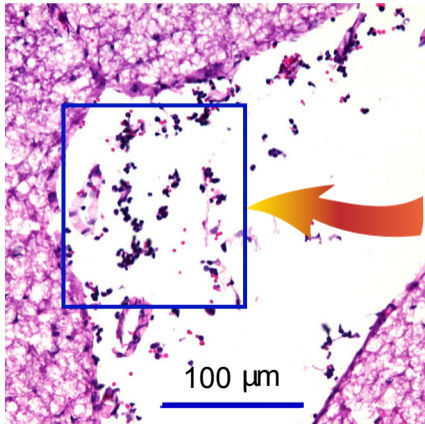

**B** No Transplant Control

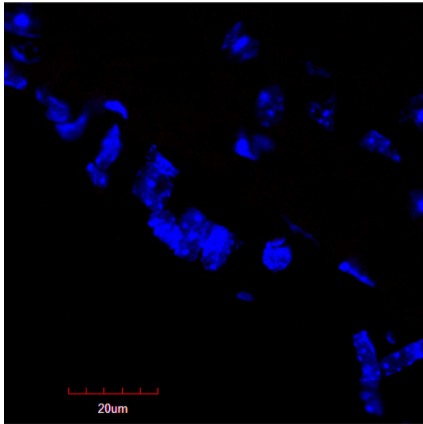

**C** 3° Transplant (Pt 11)

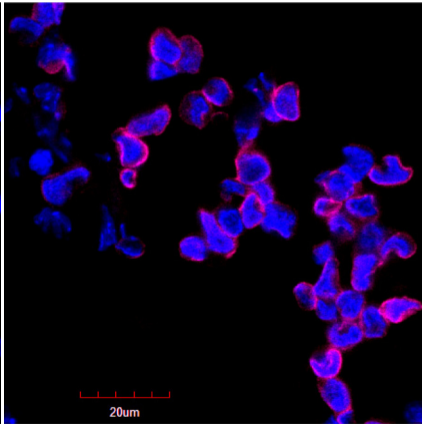

Supplement: Figure S6 — Human cell infiltration in the tertiary CD34+CD2+CD7+ cells transplanted mouse brain. 30 000 CD34+CD38+CD2+CD7+Lin− cells sorted from T-ALL patient 11 with the aid of FACS were intrahepatically transplanted into neonatal RAG2−/−gc −/− mice, and serial transplantation were done by 50 000 mouse BM cells. Tertiary transplanted mouse brain was fixed in 4% PFA and 30% sucrose overnight, separately, and then embedded in OCT for section with the thickness of 16 µm. Mouse brain sections were stained with anti-human CD45 antibody, mounted with prolong gold (Invitrogen). Images were taken under the Fluoview FVi10 confocal microscope (Olympus). (A) H & E staining of the mouse ventricular area. Scale bar is 100 µm. (B) Human CD45 Immunostaining of no transplant control mouse brain. DAPI detects the mouse cell nuclei. Scale bar is 20 µm. (C) Human CD45 Immunostaining of 3° transplant mouse brain (patient 11). Pink cell surface staining represents the human CD45, and the blue staining is the cell nuclei. Scale bar is 20 µm. (PDF) [file pone.0039725.s006.pdf]
